# Relationship between dietary carotenoid intake and sleep duration in American adults: a population-based study

**DOI:** 10.1186/s12937-023-00898-x

**Published:** 2023-12-08

**Authors:** Ming-Gang Deng, Fang Liu, Kai Wang, Yuehui Liang, Jia-Qi Nie, Jiewei Liu

**Affiliations:** 1 Department of Psychiatry, Wuhan Mental Health Center, Wuhan, Hubei 430012 China; 2 Department of Psychiatry, Wuhan Hospital for Psychotherapy, Wuhan, 430012 Hubei China; 3 School of Public Health, Wuhan University, Wuhan, 430071 Hubei China; 4 Department of Public Health, Wuhan Fourth Hospital, Wuhan, 430000 Hubei China; 5 Xiaogan Center for Disease Control and Prevention, Xiaogan, 432000 Hubei China

**Keywords:** Carotenoids, Sleep duration, American adults, NHANES

## Abstract

**Objective:**

To investigate the relationship between dietary carotenoid intake and sleep duration.

**Methods:**

Adults enrolled in the National Health and Nutrition Examination Survey (NHANES) 2007–2018 without missing information on dietary carotenoid intake (*α*-carotene, *β*-carotene, *β*-cryptoxanthin, lycopene, and lutein + zeaxanthin), sleep duration, and covariates were included. Participants’ carotenoid consumption was divided into three groups by quartiles and sleep duration was grouped as short (< 7 h/night), optimal (7–8 h/night), and long (> 8 h/night). Multinominal logistic regression was constructed to examine the association between dietary carotenoid intake and sleep duration. Restricted cubic spline (RCS) regression was further utilized to explore their dose-response relationship. The weighted quantile sum (WQS) model was adopted to calculate the mixed and individual effect of 5 carotenoid sub-types on sleep duration.

**Results:**

Multinominal logistic regression presented that people with higher intakes of *α*-carotene, *β*-carotene, *β*-cryptoxanthin, lycopene, and lutein + zeaxanthin were less likely to sleep too short or too long. Consistent with the findings from multinominal logistic regression, the RCS models suggested a reverse U-shaped relationship between sleep duration and carotenoid intakes. The mixed effects were also significant, where *β*-cryptoxanthin and lutein + zeaxanthin were the top 2 contributors associated with the decreased risks of short sleep duration, while *β*-carotene, *α*-carotene, and *β*-cryptoxanthin were the main factors related to the lower risk of long sleep duration.

**Conclusion:**

Our study revealed that the American adults with optimal sleep duration were associated with more dietary carotenoid intake, in comparison to short or long sleepers.

**Supplementary Information:**

The online version contains supplementary material available at 10.1186/s12937-023-00898-x.

## Introduction

Sleep was a naturally recurring state of mind and body, which was characterized by lowered consciousness, relatively inhibited sensory activity, inhibition of nearly all voluntary muscles, and reduced interactions with surroundings [1]. A healthy sleep pattern, composed of sleeping 7–8 h/day, early chronotype, reporting never or rare insomnia symptoms, no self-reported snoring, and no frequent daytime sleepiness, was essential to maintain physical and mental health [2, 3].

Based on the optimal sleep duration of 7–8 h/day, short and long sleep duration were generally defined as usually sleeping less than 7 h/day and usually sleeping more than 8 h/day, respectively [4–6]. Along with the development of modern society and the changes in the socioeconomic environment and lifestyle, reduced sleep duration and sleep quality have become widespread [7]. Meanwhile, long sleep duration was prevalent in several developed countries, including Australia, Finland, Germany, etc. [8]. Both short and long sleep periods have been confirmed to not only elevate the risks of developing chronic diseases including hypertension [9], type 2 diabetes [10], and depression [11], but also increase all-cause mortality [12, 13].

Many researchers have tried to investigate the causes and risk factors for abnormal sleep duration from various aspects to improve sleep health and prevent sleep-related adverse outcomes. Nutrition as an important lifestyle factor has attracted special interest and has been linked with sleep duration and other traits in many epidemiological studies [14–16]. Our previous study discovered that greens, vegetables, and fruits contributed a large proportion to the inverse relationship between healthy eating patterns and the risk of sleep disorders [17].

Carotenoids are a group of orange, yellow, or red lipid-soluble pigments abundant in greens, vegetables, and fruits [18], and approximately 80–90% of carotenoid intake in humans comes from these foods [19]. Many biological benefits of carotenoids including anti-oxidation, anti-inflammation, and immunity enhancement [20], were potentially involved in the pathology of sleep disorders [21, 22]. The previous study indicated that elevated dietary carotenoid consumption was related to lower risks of difficulty falling asleep [23], and poor sleep quality [24].

Despite this, the relevant studies were relatively limited and whether carotenoid intake was associated with sleep duration remains unclear. Therefore, we intend to investigate the relationship between dietary carotenoid intake and sleep duration, and we hypothesize that individuals with higher dietary carotenoid intake are correlated with a lower risk of abnormal sleep duration.

## Materials and methods

### Data source and participants

The data sets used in this research were retrieved from the 2007–2018 National Health and Nutrition Examination Survey (NHANES). NHANES is an ongoing, biennial, nationally representative series of surveys, which adopt a complex, multistage, probability sampling design to monitor the health and nutritional status of adults and children in the United States. Detailed information on the NHANES could be assessed at https://www.cdc.gov/nchs/nhanes/index.htm.

The protocols for NHANES were approved by the National Center for Health Statistics (NCHS) Research Ethics Review Board, and informed consent was obtained from all participants, available online at https://cdc.gov/nchs/nhanes/irba98.htm. According to 45 CFR Part 46, ethical approval and informed consent were not required for the current study as the data sets were all publicly available from NHANES.

Participants included in this study need to satisfy the following inclusion criteria: adults (aged ≥18 years old), with complete information on their sleep duration and two 24-h dietary carotenoid intakes. Those with missing data on any covariates including demographic, behavioral, and health characteristics would be excluded.

### Assessment of sleep duration

Sleep habits and disorders related questions were asked, in the home, by trained interviewers using the Computer-Assisted Personal Interviewing (CAPI) system, where consistency checks were built in to reduce data entry errors for quality assurance and control. Sleep duration data reflected self-reported usual sleep were asked “How much sleep do you usually get at night on weekdays or workdays?”. We categorized the participants into three groups: short sleep duration (< 7 h/night), optimal sleep duration (7–8 h/night), and long sleep duration (> 8 h/night) according to the previous studies [4–6], while the answers of “Do not know” and “Refused” were considered missing and omitted.

### Assessment of dietary carotenoid intake

Dietary intake information from NHANES participants was obtained from the two 24-h dietary interviews, which were conducted by trained dietary interviewers. The first dietary recall interview was collected in-person in the Mobile Examination Center (MEC) and the second was collected via telephone approximately 3 to 10 days after the first interview. Dietary carotenoid intakes used in this study including *α*-carotene (mcg/day), *β*-carotene (mcg/day), *β*-cryptoxanthin (mcg/day), lycopene (mcg/day), and lutein + zeaxanthin (mcg/day) were retrieved from the two 24-hour dietary recall interviews, and divided into three categories based on the quartiles of average amount from the two recalls. The cut-off values for each can be found in Supplementary Table S1.

### Assessment of covariates

The covariates of three dimensions, including sociodemographic, behavioral, and health characteristics, were regarded as potential confounding factors a priori.

Sociodemographic characteristics comprised age groups (18–39, 40–59, and ≥ 60 years old), sex (Female and Male), race (Non-Hispanic White, Mexican American, Non-Hispanic Black, and Other/multiracial), highest education degree (Less than high school graduate, High school graduate or GED, and Some college or above), and family income level (0–130, 130–350%, and > 350% PIR, PIR refers to the ratio of family income to poverty threshold).

Behavioral variables consisted of smoking status (Never, former, and current), drinking (Yes or No), physical activity [Inactive (< 600 metabolic equivalents of task (MET) per week) and active (≥ 600 MET/week)], and the amount of caffeine consumption per day [<Q_1_ (< 41.5 mg/day), Q_1_-Q_3_ (41.5–240 mg/day), and > Q_3_ (> 240 mg/day)].

Health characteristics included body mass index (BMI) categories [underweight/normal (≤ 24.9 kg/m^2^), overweight (25.0–29.9 kg/m^2^), and obese (≥ 30.0 kg/m^2^)], hypertension (Yes or No), diabetes (Yes or No), and depression (Yes or No).

### Statistical analysis

As suggested by the analytic guidelines of NHANES, primary sampling units (SDMVPSU), stratification (SDMVSTRA), and sampling weight (WTMEC2YR, full sample 2-year MEC exam weight) were incorporated in all analyses to generate nationally representative estimates.

Dietary carotenoid intake and potential confounding factors were summarized according to the sleep duration groups and described as the frequency with weighted percentages. Survey design-based χ^2^ tests were used to examine the associations between these variables and sleep duration phenotypes.

Since the participants were categorized into optimal, short, and long sleep duration groups, multinominal logistic regression was constructed to calculate the odds ratio (OR) and 95% confidence interval (CI) of dietary carotenoid intakes with risks among different sleep duration groups. With the people with optimal sleep duration as the reference group, the multinominal logistic regression basically worked in the same way as binary logistic regression, where the analysis broke down the sleep duration groups into two comparisons: short sleep duration vs. optimal sleep duration, and long sleep duration vs. optimal sleep duration. Additionally, to assess the confounding effects from the aforementioned three different dimensional covariates, these covariates were gradually adjusted: Model I was adjusted for sociodemographic characteristics, Model II was further adjusted for behavioral variables, and health factors were additionally added in Model III. Trend tests (*p* for trend) were performed by entering the dietary carotenoid intake (quartile-categorical) as a continuous variable and rerunning the corresponding regression models.

Furthermore, the restricted cubic spline (RCS) models were utilized to examine the dose-response relationships between dietary carotenoid intakes and sleep duration, with three knots located at the 5th, 50th, and 95th percentiles of the distributions [25, 26]. For more visual purposes, we illustrated the levels of dietary carotenoid intakes in participants with different sleep durations, with sleep duration (continuous, h/night) on the X-axis [5].

Additionally, the weighted quantile sum (WQS) regression model was used to estimate the overall mixed effects associated with five carotenoid subclasses and identify the predominant carotenoid types. Individual weight for each carotenoid was estimated using bootstrap sampling (*n* = 100), where the data were randomly split into the training set (80%) and the validation set (20%). Detailed information on the WQS regression model could be obtained from the previous literature [27].

Statistical analyses were performed in the R software 4.2.3 (R Foundation for Statistical Computing) and Stata/MP 17.0 (StataCorp, Texas, USA). All statistical tests were two-sided, and *α* = 0.05 was considered as the significance level.

## Results

**Fig. 1:**
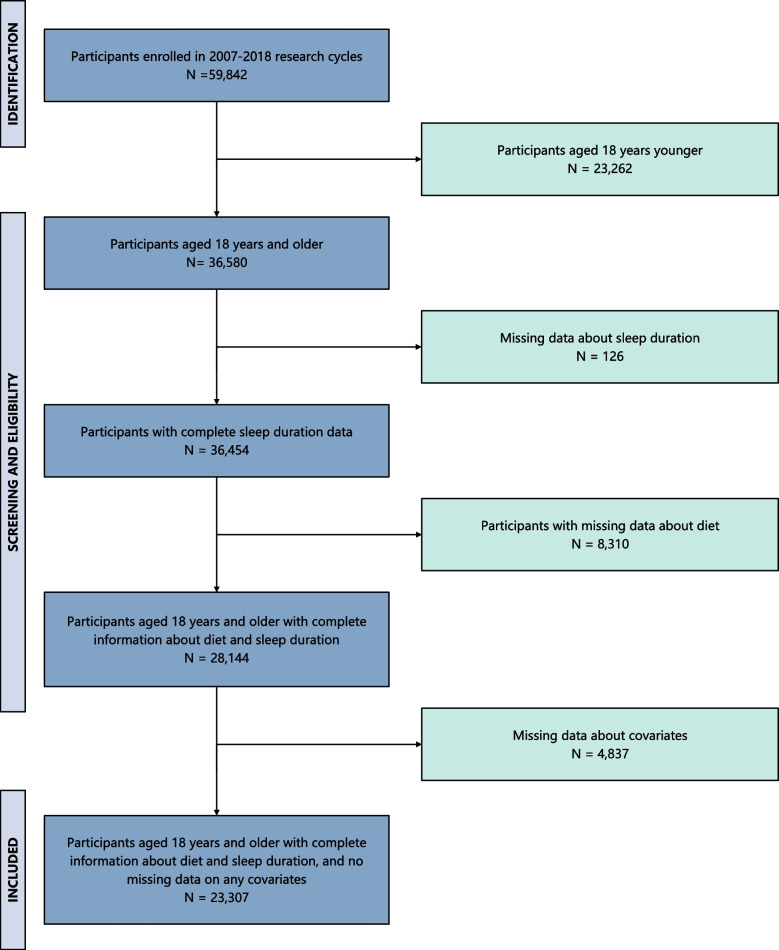
Flowchart of the population included in our final analysis

Figure 1 presents the procedures for the inclusion and exclusion of participants. A total of 59,842 people were initially recruited from 6 consecutive survey cycles of NHANES (2007–2008, 2009–2010, 2011–2012, 2013–2014, 2015–2016, and 2017–2018). After the exclusion of those aged younger than 18 years old (*N* = 23,262), without information on sleep duration (*N* = 126), with missing data about diet (*N* = 8310), and any covariates (*N* = 4837), 23,307 adults with complete information about sleep duration, diet, and covariates were finally retained. The final sample could represent non-institutionalized American adults of 166 million, with the average age being 47.5 years old and 52.17% being females.

Participants’ characteristics according to sleep duration categories were presented in Table 1. Sociodemographic, behavioral, and health characteristics distributed significantly different across different sleep duration groups, people with optimal sleep duration were more likely to be younger, female, Non-Hispanic White, with higher education and family income levels, never smoking, drinking, physically active, with moderate caffeine consumption, underweight/normal, and without comorbidities of hypertension, diabetes, and depression. Similarly, people with different sleep durations seemed to have different amounts of dietary carotenoid intake, those with optimal sleep duration were related to higher intakes of *α*-carotene, *β*-carotene, *β*-cryptoxanthin, lycopene, and lutein + zeaxanthin.

**Table 1:** Characteristics of participants across sleep duration categories

Characteristics	Optimal N = 12,460	Short *N* = 7914	Long *N* = 2933	Total *N* = 23,307	*p-value*
***Sociodemographic characteristics***					
Age (%)					< 0.001
18–39 years	4333 (35.74%)	2563 (34.79%)	1056 (38.52%)	7952 (35.77%)	
40–59 years	3952 (36.71%)	2945 (42.99%)	661 (25.31%)	7558 (37.32%)	
≥60 years	4175 (27.55%)	2406 (22.22%)	1216 (36.17%)	7797 (26.91%)	
Sex (%)					< 0.001
Female	6501 (52.46%)	3920 (48.64%)	1666 (60.1%)	12,087 (52.17%)	
Male	5959 (47.54%)	3994 (51.36%)	1267 (39.9%)	11,220 (47.83%)	
Race (%)					< 0.001
Non-Hispanic White	5927 (73.05%)	3075 (63.97%)	1344 (68.36%)	10,346 (69.7%)	
Mexican American	1855 (7.64%)	999 (7.48%)	440 (8.67%)	3294 (7.71%)	
Non-Hispanic Black	2090 (7.75%)	2232 (15.49%)	580 (10.54%)	4902 (10.46%)	
Other or multiracial	2588 (11.57%)	1608 (13.07%)	569 (12.43%)	4765 (12.13%)	
Education (%)					< 0.001
Less than high school graduate	2632 (13.08%)	1811 (15.8%)	821 (19.25%)	5264 (14.64%)	
High school graduate or GED	2653 (20.46%)	1959 (25.92%)	751 (26.32%)	5363 (22.83%)	
Some college or above	7175 (66.46%)	4144 (58.28%)	1361 (54.42%)	12,680 (62.54%)	
Income (%)					< 0.001
0 ~ 130% PIR	3462 (17.27%)	2623 (23.21%)	1145 (29.49%)	7230 (20.53%)	
130% ~ 350% PIR	4687 (34.52%)	3000 (36.7%)	1136 (37.69%)	8823 (35.56%)	
350% PIR	4311 (48.21%)	2291 (40.09%)	652 (32.81%)	7254 (43.92%)	
***Behavioral characteristics***					
Smoke (%)					< 0.001
Never	7283 (59.01%)	4186 (51.78%)	1638 (55.61%)	13,107 (56.39%)	
Former	3124 (25.96%)	1868 (24.47%)	722 (24.27%)	5714 (25.3%)	
Current	2053 (15.02%)	1860 (23.75%)	573 (20.13%)	4486 (18.31%)	
Drink (%)					0.008
No	3129 (19.31%)	2028 (20.61%)	766 (22.13%)	5923 (20.04%)	
Yes	9331 (80.69%)	5886 (79.39%)	2167 (77.87%)	17,384 (79.96%)	
Physical activity (%)					< 0.001
Inactive	3752 (24.14%)	2552 (27.07%)	1167 (33.78%)	7471 (26.17%)	
Active	8708 (75.86%)	5362 (72.93%)	1766 (66.22%)	15,836 (73.83%)	
Caffeine (%)					< 0.001
<Q_1_	3747 (24.98%)	2385 (23.92%)	993 (28.56%)	7125 (25.07%)	
Q_1_-Q_3_	6332 (49.95%)	3945 (49.37%)	1499 (51.83%)	11,776 (49.99%)	
>Q_3_	2381 (25.07%)	1584 (26.71%)	441 (19.61%)	4406 (24.94%)	
***Health characteristics***					
Body mass index (%)					< 0.001
Underweight/Normal	3633 (29.91%)	1933 (24.66%)	841 (29.51%)	6407 (28.24%)	
Overweight	4114 (32.93%)	2517 (32.49%)	920 (31.72%)	7551 (32.65%)	
Obese	4713 (37.16%)	3464 (42.85%)	1172 (38.77%)	9349 (39.1%)	
Hypertension (%)					< 0.001
No	8253 (70.12%)	4833 (65.46%)	1791 (63.56%)	14,877 (67.92%)	
Yes	4207 (29.88%)	3081 (34.54%)	1142 (36.44%)	8430 (32.08%)	
Diabetes (%)					< 0.001
No	10,731 (89.19%)	6598 (86.7%)	2394 (85.61%)	19,723 (88.01%)	
Yes	1729 (10.81%)	1316 (13.3%)	539 (14.39%)	3584 (11.99%)	
Depression (%)					< 0.001
No	11,860 (95.85%)	7060 (90.18%)	2676 (91.87%)	21,596 (93.64%)	
Yes	600 (4.15%)	854 (9.82%)	257 (8.13%)	1711 (6.36%)	
***Dietary carotenoid intake***					
*** α*-Carotene (%)**					< 0.001
<Q_1_	2939 (22.99%)	2184 (27.64%)	886 (30.39%)	6009 (25.29%)	
Q_1_-Q_3_	6321 (50.38%)	3926 (49.22%)	1417 (48.15%)	11,664 (49.76%)	
>Q_3_	3200 (26.62%)	1804 (23.14%)	630 (21.47%)	5634 (24.95%)	
*** β*-Carotene (%)**					< 0.001
<Q_1_	3047 (22.28%)	2344 (28.16%)	931 (30.12%)	6322 (25%)	
Q_1_-Q_3_	6250 (50.46%)	3749 (49.17%)	1424 (49.95%)	11,423 (50%)	
>Q_3_	3163 (27.26%)	1821 (22.67%)	578 (19.93%)	5562 (24.99%)	
*** β*-Cryptoxanthin (%)**					< 0.001
<Q_1_	2954 (24.21%)	2231 (27.78%)	838 (28.34%)	6023 (25.79%)	
Q_1_-Q_3_	6051 (49.06%)	3795 (49.9%)	1403 (49.23%)	11,249 (49.34%)	
>Q_3_	3455 (26.73%)	1888 (22.32%)	692 (22.43%)	6035 (24.87%)	
Lycopene (%)					< 0.001
<Q_1_	3216 (23.21%)	2322 (27.04%)	925 (28.52%)	6463 (25.01%)	
Q_1_-Q_3_	6287 (51.39%)	3769 (47.87%)	1360 (48.72%)	11,416 (49.99%)	
>Q_3_	2957 (25.4%)	1823 (25.09%)	648 (22.76%)	5428 (25%)	
Lutein + Zeaxanthin (%)					< 0.001
<Q_1_	3089 (22.55%)	2313 (28.49%)	912 (28.83%)	6314 (25.11%)	
Q_1_-Q_3_	6290 (50.07%)	3943 (49.26%)	1452 (50.64%)	11,685 (49.89%)	
>Q_3_	3081 (27.38%)	1658 (22.24%)	569 (20.53%)	5308 (25%)	

PIR: family income to the poverty threshold; Q1, 25^th^ percentile﻿; Q3, 75^th^ percentile

The results of multinomial logistic regression are presented in Table 2. After full adjustment for sociodemographic, behavioral, and health characteristics with people with optimal sleep duration as the reference group, those with higher intakes of *α*-carotene (OR: 0.885, 95% CI: 0.788–0.994, *p* = 0.039), *β*-carotene (OR: 0.827, 95% CI: 0.748–0.916, *p* < 0.001), *β*-cryptoxanthin (OR: 0.824, 95% CI: 0.734–0.925, *p* = 0.001), lycopene (OR: 0.847, 95% CI: 0.761–0.942, *p* = 0.002), and lutein + zeaxanthin (OR: 0.789, 95% CI: 0.712–0.874, *p* < 0.001) were less likely to be short sleepers. Additionally, long sleepers tended to consume less *α*-carotene (OR: 0.700, 95% CI: 0.602–0.815, *p* < 0.001), *β*-carotene (OR: 0.654, 95% CI: 0.566–0.757, *p* < 0.001), *β*-cryptoxanthin (OR: 0.779, 95% CI: 0.664–0.912, *p* = 0.002), lycopene (OR: 0.846, 95% CI: 0.744–0.962, *p* = 0.011), and lutein + zeaxanthin (OR: 0.753, 95% CI: 0.634–0.894, *p* = 0.001) than individuals with optimal sleep duration.

**Table 2:** Associations of dietary carotenoid intake with the risk of short and long sleep duration^a^

	Model I				Model II				Model III			
	Short sleep duration		Long sleep duration		Short sleep duration		Long sleep duration		Short sleep duration		Long sleep duration	
	OR (95% CI)	*p*_-value_	OR (95% CI)	*p*_-value_	OR (95% CI)	*p*_-value_	OR (95% CI)	*p*_-value_	OR (95% CI)	*p*_-value_	OR (95% CI)	*p*_-value_
**α-Carotene**		**0.001**		**< 0.001**		**0.014**		**< 0.001**		**0.041**		**< 0.001**
< Q_1_	ref	–	ref	–	ref	–	ref	–	ref	–	ref	–
Q_1_ - Q_3_	0.873 (0.789, 0.967)	0.009	0.763 (0.672, 0.867)	< 0.001	0.903 (0.814, 1.002)	0.053	0.782 (0.688, 0.888)	< 0.001	0.915 (0.825, 1.014)	0.087	0.783 (0.689, 0.889)	< 0.001
> Q_3_	0.823 (0.733, 0.925)	0.001	0.680 (0.585, 0.790)	< 0.001	0.865 (0.772, 0.970)	0.013	0.698 (0.600, 0.812)	< 0.001	0.885 (0.788, 0.994)	0.039	0.700 (0.602, 0.815)	< 0.001
***β***_-Carotene_		< 0.001		**< 0.001**		**< 0.001**		**< 0.001**		**< 0.001**		**< 0.001**
< Q_1_	ref	–	ref	–	ref	–	ref	–	ref	–	ref	–
Q_1_ - Q_3_	0.852 (0.778, 0.934)	0.001	0.813 (0.723, 0.915)	0.001	0.879 (0.803, 0.963)	0.006	0.827 (0.734, 0.932)	0.002	0.895 (0.817, 0.980)	0.017	0.834 (0.740, 0.939)	0.003
> Q_3_	0.757 (0.683, 0.840)	< 0.001	0.627 (0.543, 0.724)	< 0.001	0.801 (0.725, 0.886)	< 0.001	0.648 (0.560, 0.749)	< 0.001	0.827 (0.748, 0.916)	< 0.001	0.654 (0.566, 0.757)	< 0.001
***β***_-Cryptoxanthin_		**< 0.001**		**< 0.001**		**< 0.001**		**0.002**		**0.001**		**0.002**
< Q_1_	ref	–	ref	–	ref	–	ref	–	ref	–	ref	–
Q_1_ - Q_3_	0.922 (0.836, 1.017)	0.105	0.893 (0.791, 1.008)	0.068	0.893 (0.791, 1.008)	0.068	0.908 (0.805, 1.025)	0.118	0.951 (0.862, 1.049)	0.309	0.909 (0.805, 1.026)	0.120
> Q_3_	0.767 (0.684, 0.860)	< 0.001	0.755 (0.646, 0.883)	0.001	0.755 (0.646, 0.883)	0.001	0.774 (0.660, 0.909)	0.002	0.824 (0.734, 0.925)	0.001	0.779 (0.664, 0.912)	0.002
**Lycopene**		**0.059**		**0.044**		**0.102**		**0.062**		**0.160**		**0.073**
< Q_1_	ref	–	ref	–	ref	–	ref	–	ref	–	ref	–
Q_1_ - Q_3_	0.835 (0.751, 0.928)	0.001	0.831 (0.730, 0.946)	0.006	0.841 (0.757, 0.935)	0.002	0.841 (0.738, 0.957)	0.009	0.847 (0.761, 0.942)	0.002	0.846 (0.744, 0.962)	0.011
> Q_3_	0.890 (0.790, 1.002)	0.053	0.837 (0.703, 0.997)	0.046	0.901 (0.798, 1.018)	0.094	0.848 (0.712, 1.010)	0.064	0.914 (0.809, 1.033)	0.149	0.854 (0.717, 1.016)	0.075
**Lutein + Zeaxanthin**		**< 0.001**		**< 0.001**		**< 0.001**		**0.001**		**< 0.001**		**0.001**
< Q_1_	ref	–	ref	–	ref	–	ref	–	ref	–	ref	–
Q_1_ - Q_3_	0.835 (0.761, 0.917)	< 0.001	0.870 (0.765, 0.988)	0.033	0.861 (0.785, 0.945)	0.002	0.889 (0.783, 1.010)	0.070	0.868 (0.788, 0.955)	0.004	0.893 (0.786, 1.014)	0.081
> Q_3_	0.723 (0.651, 0.802)	< 0.001	0.712 (0.602, 0.843)	< 0.001	0.761 (0.688, 0.843)	< 0.001	0.742 (0.625, 0.881)	0.001	0.789 (0.712, 0.874)	< 0.001	0.753 (0.634, 0.894)	0.001

OR, odds ratio; 95% CI, 95% confidence interval; Q1, 25^th^ percentile﻿; Q3, 75^th^ percentile; Model I: Adjusted for sociodemographic characteristics. Model II: Adjusted for sociodemographic and behavioral characteristics. Model III: Adjusted for sociodemographic, behavioral, and health characteristics

Bold *p*-values indicate *p* for trend

^a^The estimates for short and long sleep duration in each model were respectively calculated with optimal sleep duration as the reference group

**Fig. 2:**
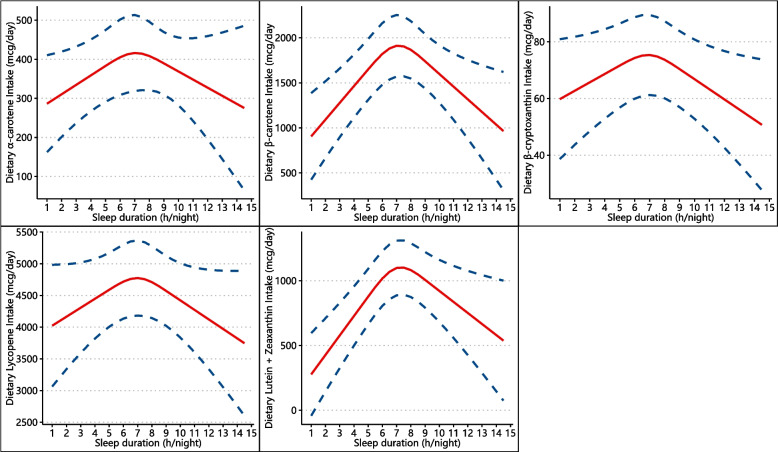
Restricted cubic spline model of sleep duration with dietary carotenoid intake. All were adjusted for sociodemographic characteristics, behavioral characteristics, and health characteristics. The red line represents the estimated dietary carotenoid intake, and the dotted blue lines represents the 95% confidence interval

The results of the dose-response associations of *α*-carotene, *β*-carotene, *β*-cryptoxanthin, lycopene, and lutein + zeaxanthin intake with sleep duration are shown in Fig. 2. After adjustment for sociodemographic, behavioral, and health characteristics, the RCS models suggested a reverse U-shaped relationship between sleep duration and carotenoid intakes. Compared to short and long sleepers, people with optimal sleep duration were presented to have more consumption of *α*-carotene, *β*-carotene, *β*-cryptoxanthin, lycopene, and lutein + zeaxanthin, which were consistent with the findings from the multinominal logistic regress models.

**Fig. 3:**
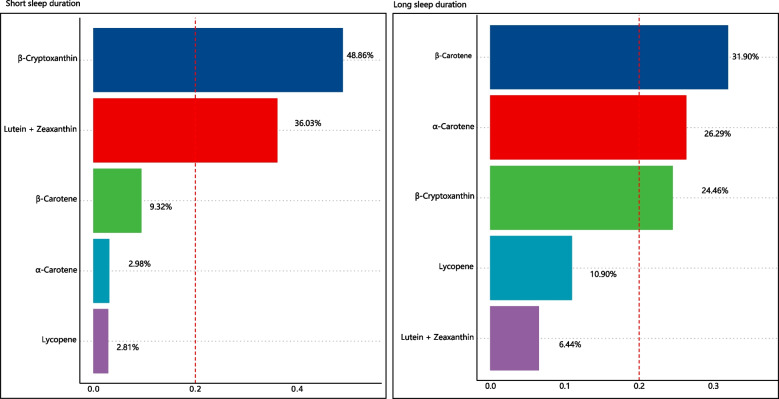
WQS model regression index weights for short/long sleep duration. The models were adjusted for sociodemographic characteristics, behavioral characteristics, and health characteristics. The dotted red line represents the assumed average effect of five carotenoid sub-classes

The mixed and individual effects of five carotenoids on sleep duration via the WQS models were presented in Fig. 3. After adjustment for sociodemographic, behavioral, and health characteristics, the overall effect of 5 carotenoids was associated with decreased risks of short (OR: 859, 95% CI: 0.796–0.928, *p* < 0.001) and long sleep duration (OR: 0.831, 95% CI: 0.739–0.932, *p* = 0.002). As for the individual effect for short sleep duration, *β*-cryptoxanthin (48.86%) and lutein + zeaxanthin (36.03%) were the top 2 important components, which exceeded the assumed average 20% effect and accounted for a total percentage of 84.89% weight. For long sleepers, *β*-carotene (31.90%), *α*-carotene (26.29%) and *β*-cryptoxanthin (24.46%) were the major components.

## Discussion

Based on the large-scale sample from six consecutive NHANES surveys, our study discovered a significant reverse U-shaped relationship between dietary carotenoid intake and sleep duration in American adults. Compared to those with short or long sleep periods, those with optimal sleep duration were correlated to a higher intake of carotenoids.

In this study, we first utilized the multinominal logistic regression to explore the association between sleep duration phenotypes and the intake of carotenoids, where the consumption of each carotenoid was categorized into three ordinal groups according to the quartiles. Generally, people with carotenoid intake in the third quartile were associated with a lower risk of sleeping too short or too long, while this did not apply to lycopene. The possible explanation is that the proportions of participants with lycopene in the third quartile among the three sleep duration groups were relatively close, thus making it insignificant after adjustments for potential confounding factors. Secondly, we discovered that carotenoid intake and sleep duration were inverse U-shaped associated via the restricted cubic spline analyses, which was consistent with the results of multinominal logistic regressions. Lastly, we evaluated the mixed effect of five carotenoid subclasses and identified the predominant types using the WQS model. The results of WQS models presented that the mixed effect of five carotenoid subclasses was also associated with lower risks of sleep too short or too long. As for the individual contributions, *β*-cryptoxanthin and lutein + zeaxanthin were the top 2 components associated with the decreased risks of short sleep duration, while *β*-carotene, *α*-carotene, and *β*-cryptoxanthin were the main factors related to the lower risk of long sleep duration. These findings were consistent with the results of multinominal logistic regression, where *β*-cryptoxanthin and lutein + zeaxanthin had the top 2 smallest OR values in the relationship to the risk of short sleep duration, and this generally applied to long sleep duration. Additionally, from the perspective of the mixed effect, *β*-cryptoxanthin was an important component associated with both short and long sleep duration, while the other carotenoids perhaps not and the underlying mechanisms need further research.

To our knowledge, only 3 studies have explored the relationship between carotenoids and sleep duration. The nutrient-wide study found that dietary lutein + zeaxanthin and lycopene intakes were associated with short and very short sleep, respectively [16]. Another study focusing on the relationships between serum nutritional biomarkers and sleep in the American population aged 20–85 discovered that lower serum total carotenoid concentration was related to higher odds of short sleep duration [28]. Plasma total carotenoids and lycopene levels were non-linear with sleep duration in 1612 UK adults aged 19–65 years [29]. The findings from our study were generally consistent with previous findings. In contrast, our study revealed that *α*-carotene, *β*-carotene, *β*-cryptoxanthin, lycopene, and lutein + zeaxanthin were all associated with the risk of both short and long sleep duration, and suggested their mixed effects were significant.

Despite limited research on carotenoids and sleep, several potential mechanisms may be appropriate to interpret our findings. A recent study found that serum pro-oxidant/antioxidant balance (PAB), which led to oxidative stress, was significantly higher in short sleepers [30]. Carotenoids may help short sleepers improve sleep duration via their prominent anti-oxidation property. Long sleep duration was associated with increases in markers of systemic inflammation [15, 22, 31, 32], while carotenoids were confirmed can impact inflammation status and reduce inflammatory response [33, 34]. In addition, carotenoids were important precursors of vitamin A, while the pre-clinical study demonstrated that vitamin A deficiency may cause sleep rhythm disturbance, especially slow-wave sleep [35]. Furthermore, higher carotenoid consumption was associated with lower risks of obesity [36], depression [37], and type 2 diabetes [38], etc., which were bidirectionally correlated to sleep duration [39, 40]. As shown in our study, the effect sizes between carotenoid intake and sleep duration were slightly attenuated after adjustment for health covariates. Lastly, fruits and vegetables were the main sources of carotenoids, and people with higher carotenoid intake tended to consume more vegetables and fruits, which to some extent reflected a healthy lifestyle [17].

Several advantages of our study were worth pointing out. We included a large sample size of 23,307 from the nationally representative survey, which enhanced the reliability and precision of our findings. In addition, three statistical models including multinominal logistic, restricted cubic spline, and weighted quantile sum regression were utilized to investigate the relationship between dietary carotenoid intake and sleep duration from three aspects, which improved the stability of our results. Furthermore, our study revealed the top contributors and the dose-response relationship between dietary carotenoid intake and sleep period, which provided new insights for further studies.

Despite these strengths, our findings should be interpreted in the context of some limitations. Firstly, the directional causality between dietary carotenoid intake and sleep duration cannot be ascertained due to the cross-sectional study design; Secondly, to improve the accuracy of dietary carotenoid intake assessment, participants without two 24-h recall data and dietary recall status being not reliable were excluded, which might induce the selection bias. Thirdly, the sleep duration was assessed via a one-item question, and the older may suffer from neurodegenerative diseases, which might affect the sleep duration assessment accuracy. Lastly, our participants were restricted to American adults, which may influence the generalization of our findings.

## Conclusion

Our study revealed that the American adults with optimal sleep duration were associated with more dietary carotenoid intake, in comparison to short or long sleepers.

### Supplementary Information

**Additional file 1: Supplement Table S1. **Grouping cut-off values for 5 kinds of carotenoids.

## Data Availability

The NHANES dataset is publicly available online, accessible at https://www.cdc.gov/nchs/nhanes/index.htm (accessed on 26 April 2023).
