# Supplementary material for: Relationship between dietary carotenoid intake and sleep duration in American adults: a population-based study
Source: Nutr J. 2023 Dec 8;22:68. doi: 10.1186/s12937-023-00898-x (PMC10704834; doi:10.1186/s12937-023-00898-x)
Supplement: Supplementary file 1 — Additional file 1: Supplement Table S1. Grouping cut-off values for 5 kinds of carotenoids. [file 12937_2023_898_MOESM1_ESM.docx]

**Supplement Table S1 Grouping cut-off values for 5 kinds of carotenoids.**

| Carotenoids | Group | Range |
| --- | --- | --- |
| α-Carotene | <Q1 | < 25 mcg/day |
|  | Q1-Q3 | 25-466 mcg/day |
|  | >Q3 | > 466 mcg/day |
| β-Carotene | <Q1 | < 472 mcg/day |
|  | Q1-Q3 | 472-2850 mcg/day |
|  | >Q3 | > 2850 mcg/day |
| β-Cryptoxanthin | <Q1 | < 14 mcg/day |
|  | Q1-Q3 | 14-95 mcg/day |
|  | >Q3 | > 95 mcg/day |
| Lycopene | <Q1 | < 844 mcg/day |
|  | Q1-Q3 | 844-6877 mcg/day |
|  | >Q3 | > 6877 mcg/day |
| Lutein + Zeaxanthin | <Q1 | < 472 mcg/day |
|  | Q1-Q3 | 472-1703 mcg/day |
|  | >Q3 | > 1703 mcg/day |

Q_1_, 25^th^ percentile; Q_3_, 75^th^ percentile.
